# Bat-associated coronavirus found in pigs in the Iberian Peninsula: insights into potential cross-species transmission

**DOI:** 10.1007/s11259-025-10794-w

**Published:** 2025-06-14

**Authors:** Sérgio Santos-Silva, Andreia V. S. Cruz, João R. Mesquita

**Affiliations:** 1 School of Medicine and Biomedical Sciences (ICBAS), University of Porto, Porto, 4050-313 Portugal; 2 Centro de Estudos de Ciência Animal (CECA), Instituto de Ciências, Tecnologias e Agroambiente (ICETA), Universidade do Porto (UP), Rua D. Manuel II, Apartado 55142, Porto, 4051-401 Portugal; 3 Associate Laboratory for Animal and Veterinary Science (AL4AnimalS), Lisbon, 1300-477 Portugal

**Keywords:** Coronavirus, One health, Swine, Zoonosis, Epidemiology

## Abstract

Coronaviruses (CoVs) are a diverse group of RNA viruses that affect both human and animal health. Swine populations are particularly relevant in the ecology of CoVs, acting as potential intermediate hosts for zoonotic transmission. This study aimed to assess the occurrence of CoVs in farmed pigs in Portugal and Spain. A total of 400 fecal samples were collected from pigs in northern Portugal and northern Spain and screened using a broad-spectrum pan-CoV nested RT-PCR assay. Of these, 18 samples (4.5%) tested positive for CoV, and phylogenetic analyses confirmed their classification within the *Alphacoronavirus* genus. The detected sequences shared high nucleotide identity with bat-associated Alphacoronaviruses from Portugal, Spain, Italy, and the United Kingdom, emphasizing the importance of continued research on the role of bats in the transmission cycle and the zoonotic potential of Alphacoronaviruses. These findings highlight the importance of ongoing surveillance in swine populations to monitor emerging CoV strains and assess potential zoonotic risks.

## Introduction

Coronaviruses (CoVs) are a diverse group of RNA viruses that have significant implications for human and animal health, particularly in domestic livestock, such as pigs and in natural reservoirs like bats (Drexler et al. 2010; Cui et al. 2019; Islam et al. 2021; Kenney et al. 2021; Kane et al. 2023). They belong to the family *Coronaviridae*, which is currently divided into three subfamilies: *Letovirinae*, *Orthocoronavirinae*, and *Pitovirinae* (Woo et al. 2023). These subfamilies comprise six recognized genera: *Milecovirus* (under *Letovirinae*), *Alphacoronavirus*, *Betacoronavirus*, *Gammacoronavirus*, *Deltacoronavirus* (all under *Orthocoronavirinae*), and *Alphapironavirus* (under *Pitovirinae*). Alphacoronaviruses include several porcine coronaviruses of veterinary relevance, notably transmissible gastroenteritis virus (TGEV) and porcine respiratory coronavirus (PRCV) (Turlewicz-Podbielska and Pomorska-Mól 2021). Members of the *Orthocoronavirinae* subfamily have been linked to major outbreaks, including the SARS and MERS epidemics, the COVID-19 pandemic, and numerous other pathogens of clinical, veterinary, and economic significance (Woo et al. 2009; Letko et al. 2020; Jacob Machado et al. 2021). The ability of CoVs to infect a wide range of hosts, including mammals and birds, underscores their potential for zoonotic transmission and the emergence of new viral strains (Edwards et al. 2020; Kenney et al. 2021; Liu et al. 2023).

Bats are widely acknowledged as evolutionary reservoirs for Alphacoronaviruses and Betacoronaviruses, serving as natural hosts due to their unique immune responses and ecological adaptability (Drexler et al. 2012; Banerjee et al. 2019; Cui et al. 2019). Studies in Portugal have confirmed the presence of genus *Alphacoronavirus*, subgenus *Pedacovirus* and *Tegacovirus*, in both cavernicolous (cave-dwelling) and tree-dwelling bats (Hemnani et al. 2023, 2024), supporting the notion that habitat and social behavior play roles in viral prevalence and transmission (Maganga et al. 2020). Bats contribute significantly to coronavirus evolution through recombination and spillover, facilitating cross-species transmission events (Leopardi et al. 2018).

Domestic pigs are not only susceptible to a variety of Alphacoronaviruses but also act as potential intermediate hosts, bridging wildlife reservoirs and human populations (Scarpa et al. 2021). Examples are viruses like TGEV, a classical enteric coronavirus, that historically causes severe diarrhea and high mortality in piglets (Liu and Wang 2021). However, its prevalence has significantly declined in many regions, likely due to the emergence of PRCV, a natural deletion mutant of TGEV with altered tropism for the respiratory tract (Turlewicz-Podbielska and Pomorska-Mól 2021). PRCV typically causes mild or subclinical respiratory disease and induces partial immunity against TGEV, contributing to its displacement in swine populations (Antas and Olech 2024). These viruses illustrate the dynamic adaptability of CoVs in pigs and the potential for emergence of novel strains under favorable ecological conditions.

The emergence of new porcine coronaviruses, driven by high-density farming, close human-animal contact, and interactions with wildlife reservoirs, highlights the need for ongoing surveillance (Duan et al. 2023). Although attention has recently focused on viruses like swine acute diarrhea syndrome coronavirus (SADS-CoV) in Asia (Zhou et al. 2018; Islam et al. 2021), the ecology of CoVs in European pig populations remains understudied. Understanding the diversity and prevalence of CoVs in pigs is crucial to anticipating and mitigating zoonotic risks.

Research indicates that CoVs originating in bats can spillover into pig populations, where further adaptation may occur, enhancing the risk of interspecies transmission (Zhou et al. 2018; Yang et al. 2019; Duan et al. 2023). Once established in pigs, these viruses can spread and circulate within swine populations, creating opportunities for further evolution and potential spillover into humans or other animals (Edwards et al. 2020; He et al. 2020; Thakor et al. 2022), highlighting the role of pigs as intermediates between wildlife reservoirs and human populations.

High-density farming conditions and frequent human contact with swine provide an ideal environment for viral transmission and recombination, raising the likelihood of novel CoV emergence.

Ongoing surveillance of CoVs in wildlife and domesticated animals, particularly bats and farmed pigs, is essential for understanding their ecological and evolutionary dynamics. Such monitoring is crucial for detecting emerging CoV strains and mitigating the risks of zoonotic outbreaks. Continued study of CoV distribution and transmission across various species and habitats is critical for global health preparedness.

As such, the aim of the present study was to explore the occurrence of CoVs in farmed pigs in Portugal, to better understand the potential risks of zoonotic spillover and contribute to future surveillance efforts.

## Materials and methods

### Swine fecal samples collection

**Fig. 1:**
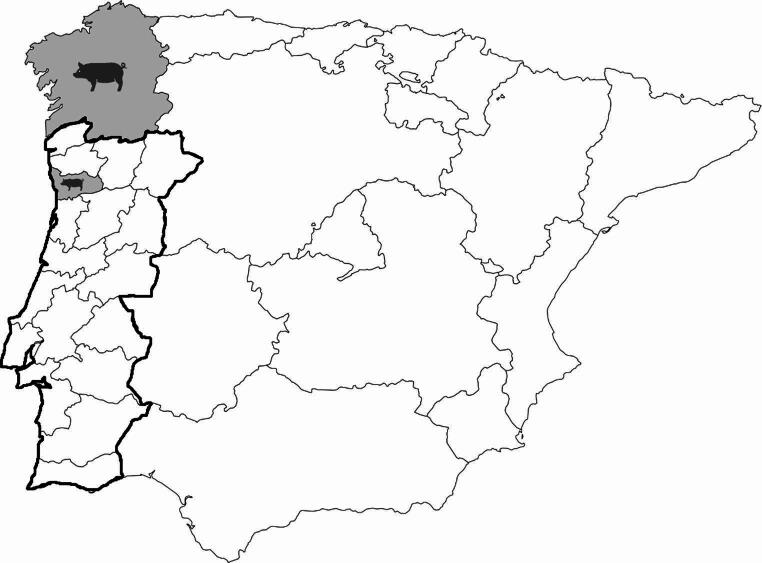
Geographic origin of swine farms included in the study, located in northern Portugal and Spain, near Porto region and Santiago de Compostela. Shaded areas and pig icons represent the approximate locations of the regions where sampled farms are situated

A total of 400 fecal samples from adult swine were obtained from a slaughterhouse in northern Portugal, which processed swine originating from five farms located in northern Portugal and Spain (Fig. 1). Of these, 200 samples were collected from swine raised on three fattening and breeding farms in northern Portugal, near the Porto region, and another 200 from two similar farms in northern Spain, near Santiago de Compostela. Sampling took place over two months, specifically in December 2021 and January 2022. Fecal samples were taken directly from the small intestine in the visceral cleaning room before the intestinal cleaning process. No animals were sacrificed specifically for this study. The samples were maintained at 4ºC and transported to the laboratory within 12 h, after which they were stored at −80ºC until nucleic acid extraction was carried out.

### Nucleic acid extraction

Fecal suspensions (10%) were prepared in phosphate-buffered saline (pH 7.2) and centrifuged at 8,000× *g* for 5 min. Nucleic acids were extracted and purified from 200 µL of the clarified supernatant using the QIAamp Viral Mini Kit (Qiagen, Hilden, Germany) on the QIAcube^®^ automated platform (Qiagen), following the protocol from the manufacturer. The RNA extracts eluted in RNase-free water were stored at − 80 °C until further analysis.

### Molecular detection of coronaviruses

The extracted RNA was analyzed for CoVs using a pan-CoV nested RT-PCR assay targeting the conserved region of the RNA-dependent RNA polymerase (*RdRp*) gene, producing a final product of 440 bp (Drzewnioková et al. 2021). A sample previously detected in our laboratory (accession number: ON721381) (Hemnani et al. 2022), was used as a positive control.

All end-point PCR reactions were conducted on a T100 thermocycler (Bio-Rad, Hercules, CA, USA). The reaction mixtures utilized the Xpert One-Step RT-PCR kit (GRiSP^®^, Porto, Portugal) for the first round and the Xpert Fast Hotstart Mastermix 2× with dye (GRiSP^®^, Porto, Portugal) for the second round. The thermocycling conditions for the first round included cDNA synthesis at 45 °C for 15 min, initial denaturation at 95 °C for 3 min, followed by 40 cycles of denaturation at 95 °C for 10 s, annealing at 50 °C for 10 s, and extension at 72 °C for 15 s, with a final extension at 72 °C for 10 min. For the second round, the initial denaturation was performed at 95 °C for 3 min, followed by 40 cycles of denaturation at 95 °C for 15 s, annealing was performed at 50 °C for 15 s, followed by extension at 72 °C for 2 s, with a final extension at 72 °C for 10 min. The amplified DNA fragments were analyzed by electrophoresis on a 1% agarose gel, stained with Xpert Green Safe DNA gel dye (GRiSP^®^, Porto, Portugal) for visualization. The gel was run at 120 V for 30 min, and a UV transilluminator was used to confirm and validate the PCR products.

### Sequencing and phylogenetic analysis

Amplicons that matched the expected size were purified using the GRS PCR & Gel Band Purification Kit (GRiSP^®^, Porto, Portugal). Following purification, bidirectional sequencing was performed using the Sanger method along with the appropriate internal primers for the target gene. The resulting sequences were aligned and analyzed using the BioEdit Sequence Alignment Editor v7.1.9 software package, and compared to sequences in the NCBI GenBank nucleotide database, retrieved on 1 July 2024 (http://blast.ncbi.nlm.nih.gov/Blast). Phylogenetic analysis was performed using MEGA X software (Kumar et al. 2018) and the Interactive Tree Of Life (iTOL) platform (Letunic and Bork 2019), incorporating the sequences from this study alongside additional reference sequences retrieved from GenBank. Reference sequences were selected based on their genetic similarity to the newly obtained sequences, inclusion of recognized coronavirus species, and representation of diverse hosts and geographic regions. Accession numbers are provided in the respective trees. The maximum-likelihood (ML) approach was used to infer the phylogenetic trees (Kumar et al. 2018), with the best-fit substitution models selected based on model testing in MEGA software. The specific models used are indicated in the corresponding figure legends. ML bootstrap values were estimated using 1,000 replicates (Tamura 1992). This model was determined by MEGA X to be the most effective replacement model.

### Statistical analysis

The detection rate of coronavirus in pigs was determined by calculating the proportion of positive samples relative to the total number of samples examined, with a 95% confidence interval (95% CI).

## Results

From a total of 400 pig fecal samples, 18 tested positive for CoV (4.5%, 95% CI: 2.69–7.02). The sequences obtained in this study have been deposited in GenBank under the accession numbers PP819529–PP819537 (isolates from Portugal) and PP977515–PP977523 (isolates from Spain). The sequences isolated from Portugal shared 95.40–99.72% identity with bat-derived isolates, including those from *Pipistrellus pipistrellus* in Portugal (OR625571), *Miniopterus schreibersii* in Spain (ON101719), *Pipistrellus pipistrellus* in Italy (OQ134959; KY780386), and *Pipistrellus pipistrellus* in the United Kingdom (OQ401253). Similarly, sequences isolated from Spain exhibited 95.90–99.76% identity with sequences from Portugal (OQ613363; OQ613361) isolated from *Rhinolophus ferrumequinum* and *Myotis myotis*, respectively. Additionally, they showed similarity to isolates from Bulgaria (GU190240) derived from *Miniopterus schreibersii*, the United Kingdom (OQ401253) from *Pipistrellus pipistrellus*, and a hedgehog-derived isolate from Portugal (OQ703961).

**Fig. 2:**
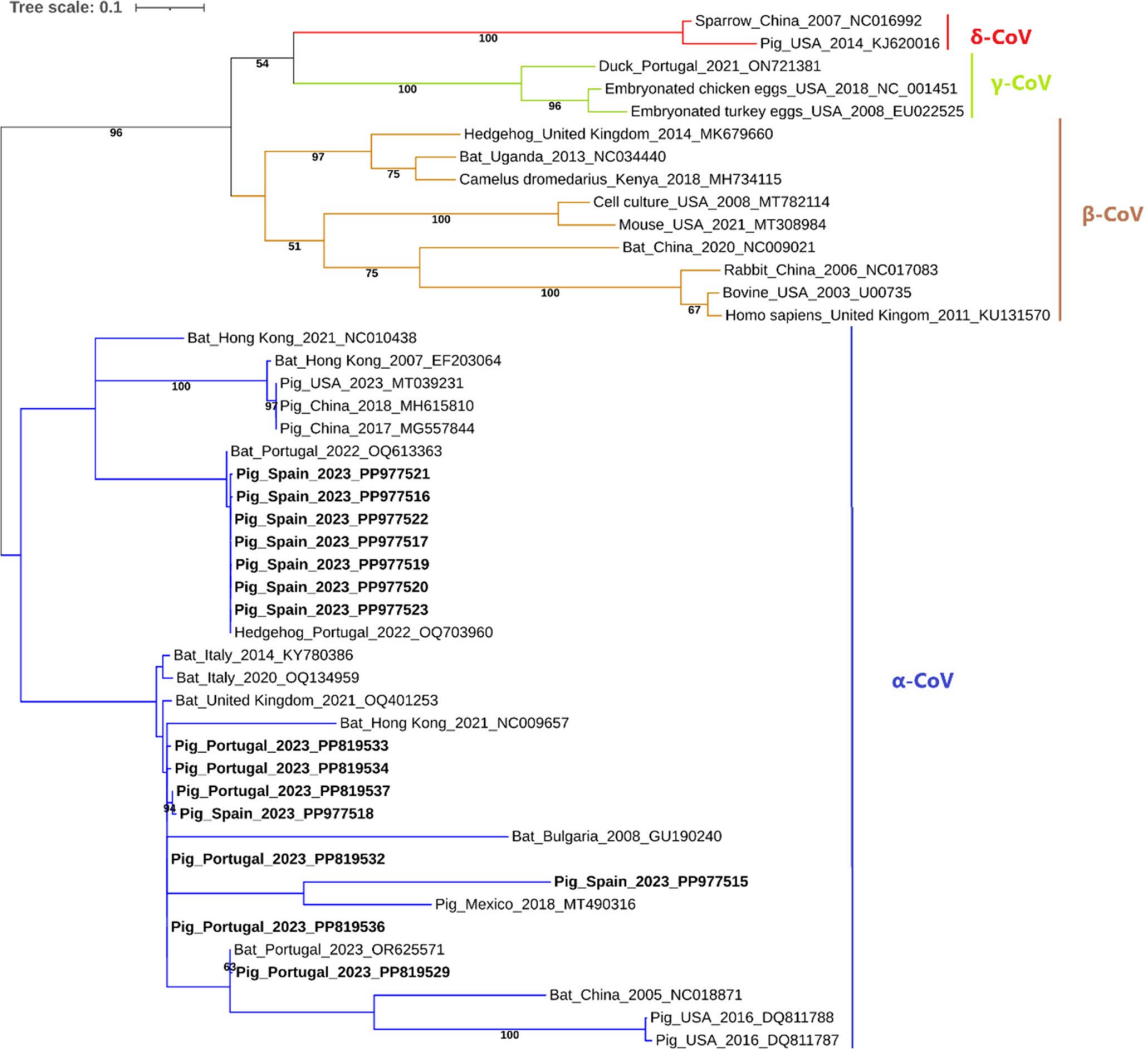
Phylogenetic tree of the *RdRp* sequences of CoV detected in this study. The tree was inferred using MEGA X software with the General Time Reversible substitution model and visualized using the Interactive Tree of Life (iTOL). The tree includes 15 sequences obtained in the present study (highlighted in bold) and 46 reference CoV nucleotide sequences retrieved from GenBank, displayed with their isolate source, location, date and accession number (without bold or shading). Only bootstrap values ≥ 50% are shown. The alignment size of the sequences was 405 base pairs

**Fig. 3:**
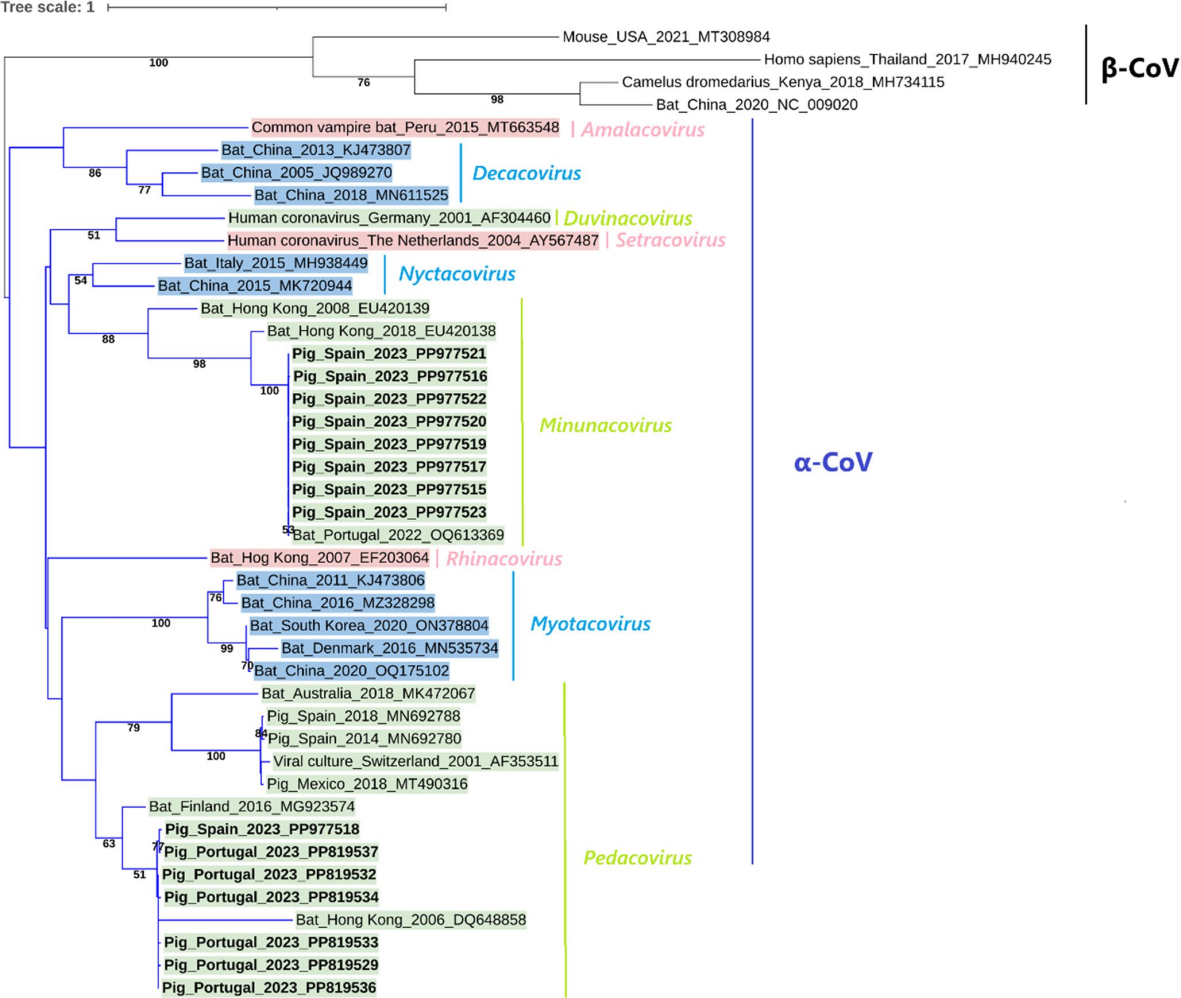
Phylogenetic tree of the *RdRp* sequences of CoV detected in this study for further subgenus characterization of *Alphacoronavirus* sequences detected. The tree was inferred using MEGA X software with the General Time Reversible substitution model and visualized using the Interactive Tree of Life (iTOL). The tree includes 15 sequences obtained in the present study (highlighted in bold) and 42 reference CoV nucleotide sequences retrieved from GenBank, displayed with their isolate source, location, date and accession number (without bold or shading). Only bootstrap values ≥ 50% are shown. The alignment size of the sequences was 366 base pairs

A phylogenetic tree was constructed using 15 sequences obtained in this study, confirming that all belonged to the *Alphacoronavirus* genus (Fig. 2). Further subdivision of the Alphacoronavirus sequences for subgenus typing (Fig. 3) suggests that sequences isolated from Spain (PP977515–PP977517 and PP977519–PP977523) belong to the subgenus *Minunacovirus*, while sequences from both Portugal and Spain (PP977518 and PP819529–PP819537) belong to the subgenus *Pedacovirus*. Additionally, a mean pairwise distance analysis was conducted to compare each obtained CoV sequence with reference CoV sequences, evaluating the number of base substitutions per site and overall genetic divergence (Table 1). Isolates B23_117 (PP819530), B23_119 (PP819535), and B23_162 (PP819531) were excluded from the analysis due to their short sequence length.

**Table 1:** The mean pairwise nucleotide identity analysis involved 27 nucleotide sequences and assessed the percentage of identical base pairs per site between sequences. The analysis focused on the mean nucleotide identity between the obtained *Alphacoronavirus* sequences and selected reference sequences. The final dataset comprised 425 bp positions. Metadata for the reference sequences includes the following: ON721381 – Avian coronavirus (chicken, China); OR625571 – *Alphacoronavirus* sp. (bat, Portugal); OQ134959, KY780386, OQ613361, OQ703961, OQ613363, and GU190240 – Bat coronaviruses (various *Pipistrellus* and *Miniopterus* spp., from Italy, Portugal, and Bulgaria); EF203064 – Bat coronavirus HKU2 (bat, China); MG557844 and MH615810 – Swine acute diarrhea syndrome coronavirus (pig, China); and OQ401253 – *Pedacovirus* (bat, United Kingdom)

Sample accession number|ID	Pairwise nucleotide identity (%)											
	ON721381	OR625571	OQ134959	KY780386	OQ613361	OQ703961	OQ613363	GU190240	EF203064	MG557844	MH615810	OQ401253
PP819529|B23_9	38.73	99.72	96.22	96.24	57.25	60.34	59.6	58.65	66.4	65.83	65.83	96.24
PP819534|B23_112	40.47	99.39	95.63	95.95	61.2	61.45	62.65	62.32	65.99	65.92	65.92	97.57
PP819536|B23_163	40.46	100	96.52	96.68	61.2	63.38	63.13	62.8	67.52	67.07	67.07	97.1
PP819532|B23_165	40.46	100	96.52	96.61	61.2	63.38	63.13	62.8	67.09	66.62	66.62	97.29
PP819537|B23_166	39.83	99.16	95.75	95.63	60.74	62.96	62.67	62.33	66.79	66.33	66.33	96.59
PP819533|B23_167	40.49	99.44	96.01	96.15	60.23	62.51	62.16	61.83	66.75	66.29	66.29	96.6
PP977515|F23_31	35.1	60.17	61.64	61.55	100	100	99.37	95.75	59.36	57.76	57.76	59.59
PP977516|F23_33	37.59	60.3	64.59	63.26	99.46	99.76	99.17	95.46	60.1	58.72	58.72	61.14
PP977517|F23_38	38.22	60.76	64.97	63.66	99.73	100	99.45	95.75	60.5	59.13	59.13	61.53
PP977518|F23_84	39.17	98.59	95.22	95.26	60.25	62.54	62.19	61.85	66.81	66.35	66.35	96.02
PP977519|F23_163	37.96	60.17	64.2	62.03	99.73	100	99.45	95.74	60.53	59.15	59.15	62.07
PP977520|F23_175	38.22	60.17	64.31	62.93	99.73	100	99.45	95.75	60.65	59.28	59.28	62.16
PP977521|F23_176	38.24	60.45	64.54	63.17	99.46	99.75	99.17	95.75	60.9	59.54	59.54	62.4
PP977522|F23_178	38.22	59.71	63.96	62.55	99.73	100	99.45	95.75	60.26	58.87	58.87	61.78
PP977523|F23_179	38.22	60.59	64.63	63.29	99.73	100	99.45	95.75	61.01	59.66	59.66	62.07

## Discussion

Coronaviruses pose a significant risk to swine health, with pigs acting as intermediate hosts for viruses originating from wildlife (Zhou et al. 2018; Thakor et al. 2022). Surveillance of swine populations is crucial for identifying CoVs with zoonotic potential and preventing future outbreaks.

In this study, we detected CoV sequences in 18 swine fecal samples, nine from Portugal and nine from Spain, with an overall detection rate of 4.5%. Furthermore, the identified sequences exhibited high nucleotide identity with bat-associated Alphacoronaviruses from various regions, including Portugal, Spain, Italy, and the United Kingdom. These findings confirm the presence of Alphacoronaviruses within swine populations in Portugal and Spain, highlighting their potential role as intermediate hosts in the transmission chain. Our phylogenetic analyses revealed that all detected CoVs belong to the *Alphacoronavirus* genus and suggest that sequences PP977515–PP977517 and PP977519–PP977523 isolated in pigs from Spain belong to the subgenus *Minunacovirus*, while sequences PP977518 isolated from a Spanish pig and PP819529–PP819537 isolated from Portuguese pigs belong to the subgenus *Pedacovirus*.

Evidence regarding the presence and impact of CoVs in European swine populations remains limited, with variations in reported detection rates potentially reflecting differences in sampling methods, geographic regions, sanitary conditions, and host densities. In our study, we detected a 4.5% occurrence rate of Alphacoronaviruses in swine, providing direct molecular evidence of their presence in Portugal. The high nucleotide identity between the detected sequences and bat-associated Alphacoronaviruses, particularly those previously identified in Portugal and Spain, supports the hypothesis that bats act as primary reservoirs and pigs as intermediate hosts, suggesting a potential spillover event in the Iberian Peninsula. In comparison, a recent serological study in southern Italy reported seroprevalence rates of 17.9% for PRCV and 1.3% for TGEV (Ferrara et al. 2023), indicating prior exposure. These methodological differences, particularly in detection approaches and viral targets, highlight the challenges in directly comparing coronavirus circulation data across studies. Nonetheless, the findings collectively support the ongoing circulation of diverse Alphacoronaviruses within European swine populations.

Moreover, previous studies have reported high sequence similarities between bat- and swine-associated CoVs, underscoring the zoonotic potential of these viruses (Edwards et al. 2020; Duan et al. 2023). Highlighting the need to explore how ecological and agricultural practices facilitate viral transmission between wildlife and livestock.

However, it is important to note that a limitation of our study is that the detection of viral RNA fragments in fecal samples does not confirm active infection in the pigs. These findings may indicate exposure or environmental contamination but do not necessarily demonstrate viral replication or systemic infection in the pigs sampled. Further studies involving serological testing and viral isolation would be needed to confirm infection status and assess transmission potential.

The detection of bat-like CoVs in swine populations underscores the importance of implementing integrated One Health approaches to mitigate zoonotic risks. The detected CoV sequences showed high nucleotide identity to bat-associated coronaviruses within the analyzed genomic region. However, given the limited length of the sequenced fragment, broader genomic similarity, particularly in key regions such as the spike gene, cannot be inferred. Despite this limitation, the presence of CoV sequences in swine highlights the potential risk of interspecies spillover, especially in areas where bats and livestock coexist or in occupational settings involving close contact with animals. Although recombination events were not directly investigated in this study, they remain a well-documented mechanism in the emergence of novel coronaviruses and warrant further genomic surveillance (Yang et al. 2021; Amoutzias et al. 2022; Nikolaidis et al. 2022).

Our findings emphasize the need for enhanced surveillance of CoVs in swine populations, particularly in regions with high bat densities, such as southern Europe (Van der Meij et al. 2015). The susceptibility of pigs to diverse CoVs, including those of bat origin, underscores the importance of targeted monitoring to track viral circulation and potential recombination events. Strengthening these efforts will be crucial for understanding cross-species transmission dynamics and mitigating future zoonotic risks.

## Conclusion

The present study provides valuable insights into the genetic relationships between swine CoVs and bat-associated Alphacoronaviruses in Europe. The high genetic similarity observed between swine CoVs and bat CoVs highlights the need for further research on the role of bats in the transmission cycle and the zoonotic potential of Alphacoronaviruses. Expanding phylogenetic analyses and investigating recombination events could shed light on the evolutionary pathways of these viruses. Comparative studies between European swine populations may help identify shared patterns of emergence and transmission.

Overall, our findings highlight the need for continuous surveillance and interdisciplinary research to better understand the dynamics of CoVs in wildlife and livestock. Such efforts are crucial to improving global health preparedness and mitigating the risks of future zoonotic outbreaks.

## Data Availability

No datasets were generated or analysed during the current study.

## References

[CR1] Amoutzias GD, Nikolaidis M, Tryfonopoulou E et al (2022) The remarkable evolutionary plasticity of coronaviruses by mutation and recombination: insights for the COVID-19 pandemic and the future evolutionary paths of SARS-CoV-2. Viruses 14. 10.3390/v14010078 10.3390/v14010078 PMC8778387 35062282

[CR2] Antas M, Olech M (2024) First report of transmissible gastroenteritis virus (TGEV) and Porcine respiratory coronavirus (PRCV) in pigs from Poland. BMC Vet Res 20. 10.1186/s12917-024-04364-6 10.1186/s12917-024-04364-6 PMC11571510 39551750

[CR3] Banerjee A, Kulcsar K, Misra V et al (2019) Bats and coronaviruses. Viruses 11:7–9. 10.3390/v11010041 10.3390/v11010041 PMC6356540 30634396

[CR4] Cui J, Li F, Shi Z-L (2019) Origin and evolution of pathogenic coronaviruses. Nat Rev Microbiol 17:181–192. 10.1038/s41579-018-0118-9 30531947 10.1038/s41579-018-0118-9 PMC7097006

[CR5] Drexler JF, Gloza-Rausch F, Glende J et al (2010) Genomic characterization of severe acute respiratory Syndrome-Related coronavirus in European bats and classification of coronaviruses based on partial RNA-Dependent RNA polymerase gene sequences. J Virol 84:11336–11349. 10.1128/jvi.00650-10 20686038 10.1128/JVI.00650-10 PMC2953168

[CR6] Drexler JF, Corman VM, Müller MA et al (2012) Bats host major mammalian paramyxoviruses. Nat Commun 3. 10.1038/ncomms1796 10.1038/ncomms1796 PMC3343228 22531181

[CR7] Drzewnioková P, Festa F, Panzarin V et al (2021) Best molecular tools to investigate coronavirus diversity in mammals: A comparison. Viruses 13. 10.3390/v13101975 10.3390/v13101975 PMC8538982 34696405

[CR8] Duan Y, Yuan C, Suo X et al (2023) Bat-Origin swine acute diarrhea syndrome coronavirus is lethal to neonatal mice. J Virol 97:1–11. 10.1128/jvi.00190-23 10.1128/jvi.00190-23 PMC10062167 36877051

[CR9] Edwards CE, Yount BL, Graham RL et al (2020) Swine acute diarrhea syndrome coronavirus replication in primary human cells reveals potential susceptibility to infection. Proc Natl Acad Sci U S A 117:26915–26925. 10.1073/pnas.2001046117 33046644 10.1073/pnas.2001046117 PMC7604506

[CR10] Ferrara G, D’Anza E, Rossi A et al (2023) A serological investigation of Porcine reproductive and respiratory syndrome and three coronaviruses in the campania region, Southern Italy. Viruses 15. 10.3390/v15020300 10.3390/v15020300 PMC9964103 36851514

[CR11] He WT, Ji X, He W et al (2020) Genomic epidemiology, evolution, and transmission dynamics of Porcine deltacoronavirus. Mol Biol Evol 37:2641–2654. 10.1093/molbev/msaa117 32407507 10.1093/molbev/msaa117 PMC7454817

[CR12] Hemnani M, Rodrigues D, Santos N et al (2022) Molecular detection and characterization of coronaviruses in migratory ducks from Portugal show the circulation of *gammacoronavirus* and *deltacoronavirus*. Anim 12(23):3283. 10.3390/ani12233283 10.3390/ani12233283 PMC9736399 36496804

[CR13] Hemnani M, da Silva PG, Thompson G et al (2023) First report of *Alphacoronavirus* Circulating in cavernicolous bats from Portugal. Viruses 15. 10.3390/v15071521 10.3390/v15071521 PMC10384150 37515207

[CR14] Hemnani M, da Silva PG, Thompson G et al (2024) Presence of *Alphacoronavirus* in Tree- and Crevice-Dwelling bats from Portugal. Viruses 16:1–12. 10.3390/v16030434 10.3390/v16030434 PMC10976264 38543799

[CR15] Islam A, Ferdous J, Islam S et al (2021) Evolutionary dynamics and epidemiology of endemic and emerging coronaviruses in humans, domestic animals, and wildlife. Viruses 13:1–27. 10.3390/v13101908 10.3390/v13101908 PMC8537103 34696338

[CR16] Jacob Machado D, Scott R, Guirales S, Janies DA (2021) Fundamental evolution of all *Orthocoronavirinae* including three deadly lineages descendent from Chiroptera-hosted coronaviruses: SARS-CoV, MERS-CoV and SARS-CoV-2. Cladistics 37:461–488. 10.1111/cla.12454 34570933 10.1111/cla.12454 PMC8239696

[CR17] Kane Y, Wong G, Gao GF (2023) Animal models, zoonotic reservoirs, and Cross-Species transmission of emerging Human-Infecting coronaviruses. Annu Rev Anim Biosci 11:1–31. 10.1146/annurev-animal-020420-025011 36790890 10.1146/annurev-animal-020420-025011

[CR18] Kenney SP, Wang Q, Vlasova A et al (2021) Naturally occurring animal coronaviruses as models for studying highly pathogenic human coronaviral disease. Vet Pathol 58:438–452. 10.1177/0300985820980842 33357102 10.1177/0300985820980842

[CR19] Kumar S, Stecher G, Li M et al (2018) MEGA X: molecular evolutionary genetics analysis across computing platforms. Mol Biol Evol 35:1547–1549. 10.1093/molbev/msy096 29722887 10.1093/molbev/msy096 PMC5967553

[CR20] Leopardi S, Holmes E, Gastaldelli M et al (2018) Interplay between co-divergence and cross-species transmission in the evolutionary history of Bat coronaviruses. Infect Genet Evol 58:279–289. 10.1016/j.meegid.2018.01.012 29355607 10.1016/j.meegid.2018.01.012 PMC7106311

[CR21] Letko M, Seifert SN, Olival KJ et al (2020) Bat-borne virus diversity, spillover and emergence. Nat Rev Microbiol 18:461–471. 10.1038/s41579-020-0394-z 32528128 10.1038/s41579-020-0394-z PMC7289071

[CR22] Letunic I, Bork P (2019) Interactive tree of life (iTOL) v4: recent updates and new developments. Nucleic Acids Res 47:256–259. 10.1093/nar/gkz239 10.1093/nar/gkz239 PMC6602468 30931475

[CR23] Liu Q, Wang H-Y (2021) Porcine enteric coronaviruses: an updated overview of the pathogenesis, prevalence, and diagnosis. Vet Res Commun 45:75–86. 10.1007/s11259-021-09808-0 34251560 10.1007/s11259-021-09808-0 PMC8273569

[CR24] Liu W, Zhang M, Hu C et al (2023) Remdesivir derivative VV116 is a potential Broad-Spectrum inhibitor of both human and animal coronaviruses. Viruses 15. 10.3390/v15122295 10.3390/v15122295 PMC10748125 38140536

[CR25] Maganga GD, Pinto A, Mombo IM et al (2020) Genetic diversity and ecology of coronaviruses hosted by cave-dwelling bats in Gabon. Sci Rep 10:1–13. 10.1038/s41598-020-64159-1 32355260 10.1038/s41598-020-64159-1 PMC7192909

[CR26] Nikolaidis M, Markoulatos P, Van De Peer Y et al (2022) The neighborhood of the Spike gene is a hotspot for modular intertypic homologous and nonhomologous recombination in coronavirus genomes. Mol Biol Evol 39. 10.1093/molbev/msab292 10.1093/molbev/msab292 PMC8549283 34638137

[CR27] Scarpa F, Sanna D, Azzena I et al (2021) Update on the phylodynamics of SADS-CoV. Life 11:1–11. 10.3390/life11080820 10.3390/life11080820 PMC8402179 34440564

[CR28] Tamura K (1992) Estimation of the number of nucleotide substitutions when there are strong transition-transversion and G + C-content biases. Mol Biol Evol 9:678–687. 10.1093/oxfordjournals.molbev.a040752 1630306 10.1093/oxfordjournals.molbev.a040752

[CR29] Thakor JC, Dinesh M, Manikandan R et al (2022) Swine coronaviruses (SCoVs) and their emerging threats to swine population, inter-species transmission, exploring the susceptibility of pigs for SARS-CoV-2 and zoonotic concerns. Vet Q 42:125–147. 10.1080/01652176.2022.2079756 35584308 10.1080/01652176.2022.2079756 PMC9225692

[CR30] Turlewicz-Podbielska H, Pomorska-Mól M (2021) Porcine coronaviruses: overview of the state of the Art. Virol Sin 36:833–851. 10.1007/s12250-021-00364-0 33723809 10.1007/s12250-021-00364-0 PMC7959302

[CR31] Van der Meij T, Van Strien AJ, Haysom KA et al (2015) Return of the Bats? A prototype indicator of trends in European Bat populations in underground hibernacula. Mamm Biol 80:170–177. 10.1016/j.mambio.2014.09.004

[CR32] Woo PCY, Lau SKP, Lam CSF et al (2009) Comparative analysis of complete genome sequences of three avian coronaviruses reveals a novel group 3c coronavirus. J Virol 83:908–917. 10.1128/JVI.01977-08 18971277 10.1128/JVI.01977-08 PMC2612373

[CR33] Woo PCY, De Groot RJ, Haagmans B et al (2023) ICTV virus taxonomy profile: *Coronaviridae* 2023. J Gen Virol 104:1–2. 10.1099/jgv.0.001843 10.1099/jgv.0.001843 PMC12135074 37097842

[CR34] Yang Y-L, Qin P, Wang B et al (2019) Broad Cross-Species infection of cultured cells by Bat HKU2-Related swine acute diarrhea syndrome coronavirus and identification of its replication in murine dendritic cells *In vivo* highlight its potential for diverse interspecies transmission. J Virol 93. 10.1128/jvi.01448-19 10.1128/JVI.01448-19 PMC6880172 31554686

[CR35] Yang Y, Yan W, Hall AB, Jiang X (2021) Characterizing transcriptional regulatory sequences in coronaviruses and their role in recombination. Mol Biol Evol 38:1241–1248. 10.1093/molbev/msaa281 33146390 10.1093/molbev/msaa281 PMC7665640

[CR36] Zhou P, Fan H, Lan T et al (2018) Fatal swine acute diarrhoea syndrome caused by an HKU2-related coronavirus of Bat origin. Nature 556:255–259. 10.1038/s41586-018-0010-9 29618817 10.1038/s41586-018-0010-9 PMC7094983

